# Supplementary material for: Laboratory Evaluation of Indigenous and Commercial Entomopathogenic Nematodes against Red Palm Weevil, Rhynchophorus ferrugineus (Coleoptera: Curculionidae)
Source: Insects. 2024 Apr 19;15(4):290. doi: 10.3390/insects15040290 (PMC11050374; doi:10.3390/insects15040290)
Supplement: Supplementary file 1 [file insects-15-00290-s001.zip › insects-2924097-supplementary.pdf]

Table S1. Detailed information about the collection of soil samples to explore indigenous entomopathogenic nematode species from different regions in the Kingdom of Saudi Arabia.

No	Province	City	Vegetation type	Soil Temp./RH	GPS
1	Al Jouf	Qariyat	*Phoenix dactylifera*	28°C/ 42%	31°17'19.6"N 37°22'20.2"E
2		Qariyat	*Phoenix dactylifera*	27°C/ 42%	31°17'52.6"N 37°21'58.2"E
3		Basita	*Olea europaea*	28°C/ 48%	30°15'50.8"N 38°14'27.9"E
4		Basita	*Olea europaea*	27°C/ 57%	30°15'50.8"N 38°14'27.9"E
5		Basita	*Olea europaea*	26°C/ 60%	30°9'38.2"N 38°20'13.6"E
6		Qariyat	*Phoenix dactylifera*	25°C/ 47%	31°16'46.2"N 37°22'20.2"E
7		Basita	*Zea mays*	26°C/ 50%	30°15'50.8"N 38°14'27.9"E
8		Qariyat	*Olea europaea*	28°C/ 40%	31°16'22.1"N 37°21'31"E
9		Qariyat	*Olea europaea*	28°C/ 54%	31°16'39.7"N 37°21'23.1"E
10	Tabuk	Qaryat Ain	*Punica granatum*	28°C/ 47%	27°38'16.5"N 36°49'14.2"E
11		An Nashifah	*Phoenix dactylifera*	28°C/ 60%	27°1'6.6"N 37°17'26.2"E
12		Qaryat Ain	*Phoenix dactylifera*	29°C/ 45%	27°38'16.5"N 36°49'14.2"E
13		Qaryat Ain	*Phoenix dactylifera*	27°C/ 45%	27°38'16.4"N 36°49'9.4"E
14		Qaryat Ain	*Phoenix dactylifera*	28°C/ 59%	27°38'16.5"N 36°49'14.2"E
15		An Nashifah	*Phoenix dactylifera*	29°C/ 60%	27°1'10.6"N 37°16'44.4"E
16		Al Wajh	*Phoenix dactylifera*	28°C/ 60%	26°14'22.9"N 36°28'53.4"E
17		Al Umlaj	*Phoenix dactylifera*	27°C/ 40%	25°05'17.2"N 37°20'6.3"E
18		Al Umlaj	*Phoenix dactylifera*	28°C/ 50%	25°01'56.9"N 37°20'6.6"E
19		Al Umlaj	*Mangifera indica*	27°C/ 50%	25°05'11.8"N 37°20'13.1"E
20		Al Umlaj	*Mangifera indica*	28°C/ 47%	25°05'6.1"N 37°20'16.4"E
21		Al Umlaj	*Mangifera indica*	27°C/ 50%	25°05'6.1"N 37°20'16.4"E
22		Al Umlaj	Lemon	28°C/ 60%	25°5'24.4"N 37°19'32.2"E
23		Al Wajah	*Phoenix dactylifera*	28°C/ 60%	26°15'11.1"N 36°31'15.7"E
24	Madinah	As Sovirqiya	*Phoenix dactylifera*	26°C/ 60%	23°20'43.4"N 40°18'30.1"E
25		Al Faraa	*Phoenix dactylifera*	25°C/ 55%	24°58'8.2"N 38°02'54.5"E
26		As Sovirqiya	*Phoenix dactylifera*	28°C/ 40%	23°19'23.3"N 40°14'55.2"E
27		As Sovirqiya	*Phoenix dactylifera*	30°C/ 43%	23°19'32.6"N 40°16'51.7"E
28		As Sovirqiya	*Phoenix dactylifera*	29°C/ 47%	23°20'43.4"N 40°18'30.1"E
29		Bir al Mashi	*Phoenix dactylifera*	30°C/ 52%	24°6'37.9"N 39°34'31.9"E
30		As Sovirqiya	*Phoenix dactylifera*	27°C/ 45%	23°19'23.3"N 40°14'55.2"E
31		As Sovirqiya	*Phoenix dactylifera*	26°C/ 47%	23°18'59.6"N 40°15'21.6E
32	Riyadh	Huraymila	*Phoenix dactylifera*	30°C/ 40%	25°07'277"N 46°06'970"E
33		Huraymila	*Phoenix dactylifera*	30°C/ 40%	25°07'307"N 46°06'110"E
